# *In situ* investigations of the phase change behaviour of tungsten oxide nanostructures

**DOI:** 10.1098/rsos.171932

**Published:** 2018-04-25

**Authors:** Kunyapat Thummavichai, Nannan Wang, Fang Xu, Graham Rance, Yongda Xia, Yanqiu Zhu

**Affiliations:** 1 College of Engineering Mathematics and Physical Sciences, University of Exeter, Exeter EX4 4QF, UK; 2 Faculty of Engineering, University of Nottingham, Nottingham NG7 2RD, UK; 3 Nanoscale and Microscale Research Centre, University of Nottingham, Nottingham NG7 2RD, UK

**Keywords:** phase transition, tungsten oxide, nanowires

## Abstract

This study uses two *in situ* techniques to investigate the geometry and phase change behaviour of bundled ultrathin W_18_O_49_ nanowires and WO_3_ nanoparticles. The *in situ* X-ray diffraction (XRD) results have shown that the phase transition of WO_3_ nanoparticles occurs in sequence from monoclinic (room temperature) → orthorhombic (350°C) → tetragonal (800°C), akin to bulk WO_3_; however, W_18_O_49_ nanowires remain stable as the monoclinic phase up to 500°C, after which a complete oxidation to WO_3_ and transformation to the orthorhombic β-phase at 550°C is observed. The *in situ* Raman spectroscopy investigations have revealed the Raman peak downshifts as the temperature increases, and have identified the 187.6 cm^−1^ as the fingerprint band for the phase transition from γ- to β-phase of the WO_3_ nanoparticle. Furthermore, WO_3_ nanoparticles exhibit the γ- to β-phase conversion at 275°C, which is about 75°C lower than the relaxation temperature of 350°C for the monoclinic γ-W_18_O_49_ nanowires. These new fundamental understandings on the phase transition behaviour offer important guidance for the design and development of tungsten oxide-based nanodevices by defining their allowed operating conditions.

## 1. Introduction

Tungsten oxides are one of the most promising transition metal oxide semiconducting materials which possess outstanding electronic, optical, chromic and sensing properties that make them suitable for a diverse range of energy-related applications [1–4]. Tungsten oxides have various interesting structural transformations, in addition to their numerous non-stoichiometric chemical compositions, hence they have attracted immense research attention. For decades, studies have been carried out to explore their different sub-stoichiometric structures and phase transformation characteristics, attempting to establish structure–property relationships and thus guide the suitability for a multitude of advanced applications, such as in solar cells, displays, microelectronic, superconductivity, photocatalytic and especially smart optical devices [1,5–8]. Bulk tungsten trioxide (WO_3_) exhibits a ReO_3_-type cubic structure (perovskite-like structure), with corner-sharing WO_6_ octahedral as the basic structure element, while bulk monoclinic W_18_O_49_ (i.e. WO_2.72_), a typical stable form of sub-stoichiometric oxide, consists of an ordered two-dimensional lattice of edge-sharing WO_6_ octahedral forming a network of pentagonal columns interspersed with hexagonal channels [9]. Under ambient pressure, the phase changes of bulk WO_3_ are temperature dependent, exhibiting a clear sequence of phase transitions with elevating temperature: from monoclinic II (ε-WO_3_, less than −43°C) to triclinic (δ-WO_3_, −43 to 17°C), to monoclinic I (γ-WO_3_, 17–330°C), to orthorhombic (β-WO_3_, 330–740°C) and, finally, to tetragonal (α-WO_3_, greater than 740°C) [10,11]. Bulk W_18_O_49_ essentially follows an analogous pattern of phase transition.

Recent developments in nanostructured materials present new opportunities and challenges for research and applications, with nanorods and nanowires of tungsten oxides convincingly outperforming their traditional bulk counterparts in the applications described previously [2,3,12,13]. However, an in-depth understanding of the phase transition characteristics of these new nanostructures has yet to be achieved, which will ultimately restrict the development of new technologies based on these novel nanomaterials. As a typically stable form of nanostructured tungsten oxide, W_18_O_49_ nanorods and nanowires have most often been reported and have stood out from other sub-stoichiometric compositions since their first synthesis a decade ago [14]. Therefore, using advanced techniques to investigate the fundamental structural features of this type of one-dimensional nanomaterial is particularly of interest.

Nanorods and nanowires of W_18_O_49_, akin to their bulk crystalline form, consist of a similar WO_6_ octahedral structure, hence could possess a variety of complex phase transitions and a temperature- and pressure-dependent phase transition sequence. Owing to the complex nature of tungsten oxides, several approaches using conventional techniques have been attempted to investigate, understand and explain the characteristics of their phase transformations. Chen *et al*. [15] studied the phase transition of WO_3_ nanowires under different hydrostatic pressure conditions, ranging from atmospheric to 42.5 GPa. Their Raman spectra have confirmed that the WO_3_ nanowires have higher phase transition pressures than their corresponding bulk WO_3_ nanocrystals. Cazzanelli [16], using both X-ray diffraction (XRD) and Raman spectroscopy to study WO_3_ and H-doped WO_3_ spherical powders, has shown that a sequence of phase transitions, from monoclinic to orthorhombic to tetragonal, has been achieved with increasing temperatures from room temperature up to 800°C. Raman spectroscopy is a more effective technique than XRD in revealing structure transitions of the complex WO_x_ system, as Raman spectroscopy has higher sensitivity to changes in the positions of and bonding between the W and O atoms in the crystal lattice, while with XRD it is often difficult to distinguish the similar and often overlapping diffraction peaks. Using Raman spectroscopy, Lai [17] has investigated the structural change of WO_3_ nanoplatelet films containing different amounts of ammonium fluoride and related it to the photocatalytic properties. Lu *et al*. [18] have reported the oxidation and phase transition of sub-stoichiometric W_18_O_49_ nanowires using intrinsic Raman spectroscopy by changing the input laser power.

The understanding of the fundamental aspects of the phase transformations of these nanostructured WO_x_ materials could unlock the mechanisms of these phase transitions, distinguish the difference between the nano and bulk forms and provide effective guidance towards the design and development of new devices. For example, the application of WO_x_ as the detecting element in electrochemical gas sensors normally involves the use of a high operating temperature to compensate for the negative effect of baseline drifting. As semiconducting materials aimed at nano-device applications, severe changes in temperature and pressure could lead to unexpected unstable performance and even failure, due to undesired phase transitions. In this paper, we report our investigations into the reversibility of the relationship between temperature and phase transition behaviour of ultrathin W_18_O_49_ nanowires, relative to spherical WO_3_ nanoparticles, by using two *in situ* hot-stage techniques, namely XRD and Raman spectroscopy, combined with *ex situ* electron microscopy analyses. This fundamental study offers the potential for phase and morphology control via temperature under different atmosphere conditions, as we believe that the temperature-associated phase and morphology changes could affect the structure transitions by way of lattice distortion, relaxation of the W–O bonding and oxygen vacancy inside the WO_x_ structures. These impacts would, therefore, influence the final performance of the nanomaterials in chromic device and sensor applications. We hope this study could serve as an important guidance for the design and optimization of future WO_x_-based devices where temperature is involved during operation.

## 2. Material and characterizations

W_18_O_49_ nanowires, approximately 2–5 nm in diameter and up to 2 µm in length, were prepared by a simple solvothermal technique by reacting WCl_6_ with cyclohexanol at 200°C for 6 h, as previously described in detail [19–21]. WO_3_ nanoparticles, approximately 40 nm in diameter, were purchased from Sigma Aldrich (UK). Both the as-purchased WO_3_ nanoparticles and the as-prepared W_18_O_49_ nanowire thin films were prepared as follows: 0.1 g dry powder was dispersed in 2 ml ethanol, which was then dispersed in an ultrasonic bath for 30 min at room temperature. After forming a homogeneous suspension, 0.6 ml of this suspension was dropcast onto a quartz substrate to form a thin film, which was then dried under room temperature overnight, prior to the *in situ* hot-stage XRD (D8 advanced) investigation, using a Cu radiation generated at 40 kV and 40 mA. The measurements were recorded in 50°C intervals, from room temperature up to 900°C under low vacuum condition. The heating rate was 50°C min^−1^, with 10 min dwell time for each step to record the diffraction profile. The scanned 2*θ* range was 20–40° for the WO_3_ and 20–45° for the W_18_O_49_ samples. The data were collected and processed using DIFFRAC.SUITE (Bruker axs 2009–2016, v.6.5.0) and DIFFRAC.EVA (Bruker axs 2010–2016, v. 4.2.0.31), respectively. The dimensional and morphological changes of the post-treated WO_3_ and W_18_O_49_ nanostructures at selected stages were observed using a HITACHI S3200N scanning electron microscope (SEM), operated at 20 kV. The JEM-1400 transmission electron microscope (TEM) operated at 200 kV was used to investigate the HRTEM and SAED image of each sample. X-ray photoelectron spectroscopy (XPS) was used to determine the different chemical compositions between WO_3_ and W_18_O_49_. The XPS study was operated via a Kratos AXIS ULTRA spectrometer with a mono-chromated Al KR X-ray source (1486.6 eV) which was operated at a 15 kV anode potential and a 10 mA emission current. The XPS data were collected and analysed using SPECTRA, v. 8.5-D-A and Casa XPS, v. 2.3.16 PR 1.6, respectively. Raman spectroscopy was conducted using a Horiba–Jobin–Yvon LabRAM HR spectrometer. Spectra were acquired using a 532 nm laser at variable power (0.01–100%, 0.00336–33.6 mW), a 50× objective and a 300 µm confocal pinhole. To simultaneously scan a range of Raman shifts, a 600 lines mm^−1^ rotatable diffraction grating along a path length of 800 mm was used. Spectra were detected using a Synapse CCD detector (1024 pixels) thermoelectrically cooled to −60°C. Before spectra collection, the instrument was calibrated using the Rayleigh line at 0 cm^−1^ and a standard Si (100) reference band at 520.7 cm^−1^. Samples were deposited onto Si (100) wafers, inserted into a Linkam LTS350 stage and the temperature profile modulated using a Linkam TMS94 temperature controller. The measurements were recorded in 25°C intervals, from room temperature up to 350°C in air. The heating rate was 10°C min^−1^, with 5 min dwell time for each step to record the spectra.

## 3. Results and discussion

**Figure 1.**
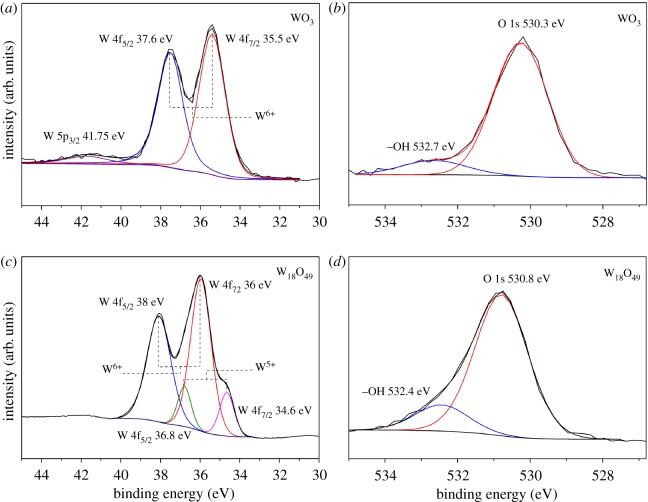
High-resolution XPS W4f and 01s spectra of WO_3_ nanoparticles (*a*,*b*) and the as-prepared W_18_O_49_ bundled nanowires (*c*,*d*) obtained at room temperature.

To understand the difference in chemical state between WO_3_ and W_18_O_49_, we acquired the XPS results, and the high-resolution W4f and O1 s XPS spectra in both samples are shown in figure 1. For the WO_3_ nanoparticle (figure 1*a*), W4f containing only W^6+^ was presented which consisted of double peaks at binding energies of 35.5 and 37.6 eV for W4f_7/2_ and W4f_5/2_, respectively. in the case of W_18_O_49_, the W4f core-level spectrum was broadened which indicates the multiple peak overlap, as shown in figure 1*c*. The separated two double peaks were associated with two different oxidation states of W atoms. The main peaks of W4f_7/2_ (36 eV) and W4f_5/2_ (38 eV) were attributed to the W^6+^ oxidation state. Another double with a lower binding energy at 34.6 and 36.8 eV was possibly caused by the emission of W4f_7/2_ and W4f_5/2_, respectively, and was assigned to the W^5+^ oxidation state. These results agreed well with previous reports [22–24]. The high-resolution O1s of both samples consisted of two peaks at 530.2 and 532.7 eV for the WO_3_ and at 530.8 and 532.4 eV for W_18_O_49_ which could be assigned to the oxygen bond with W in the structure, respectively, as shown in figure 1*b*,*d*. The shift at 532.4 O1s of W_18_O_49_ was indicative of more defects (oxygen vacancy) and weaker W–O bounding; however, the peak at 530.8 shifted towards higher binding energy compared with the WO_3_ O1s, which should be considered as evidence of the W^5+^ state inside the structure of W_18_O_49_.

**Figure 2.**
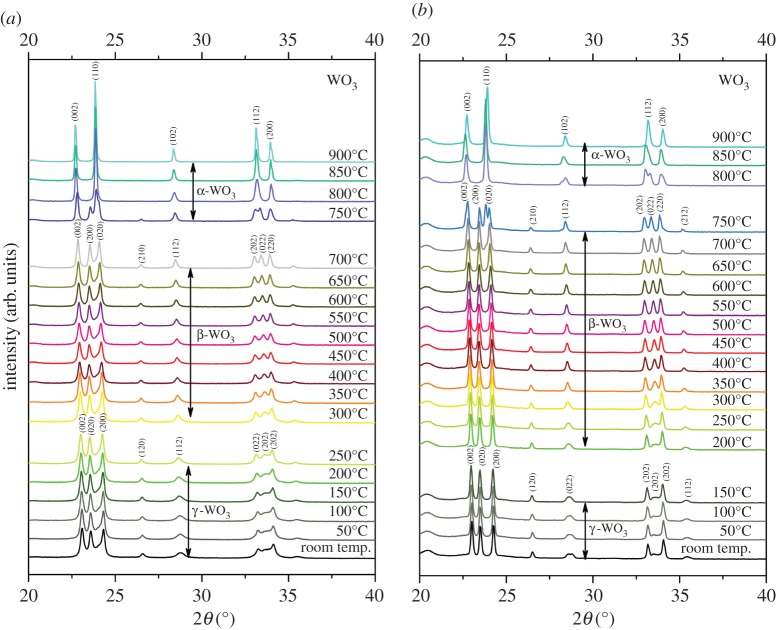
A series of *in situ* XRD profiles of the WO_3_ nanoparticles obtained during heating (*a*) and cooling (*b*), from room temperature to 900°C, in 50°C increments.

At room temperature, the XRD patterns of the as-purchased WO_3_ nanoparticle showed several main diffraction peaks at 23.1°, 23.6°, 24.3°, 26.6°, 28.8°, 33.4° and 34.1°, which were assigned to the (002), (020), (200), (120), (112), (022) and (202) planes of the monoclinic I (γ-WO_3_) phase (COD 2106382), respectively, as shown in figure 2*a*. These nanoparticles remained as the monoclinic phase until 250°C, with the orthorhombic phase (β-WO_3_, COD 2107312) detected as the temperature reached 300°C. The main 2*θ* diffraction peaks appearing at 22.9°, 23.5°, 24.2°, 26.5°, 28.6°, 33.1°, 33.6° and 34.0° were indexed as the (002), (200), (020), (210), (112), (202), (022) and (220) planes, respectively. The β-WO_3_ phase was continually identified until 700°C, then began to change to tetragonal α-WO_3_ at 750°C. As can be seen from the XRD profile (figure 2*a*), the (202) peak started to merge with the (022) peak at approximately 24° and the (200) peak also started to merge with (020) peak at approximately 33° when the temperature reached 750°C. At 800°C, the WO_3_ was completely converted to the tetragonal phase (α-WO_3_, COD 1521532), with recognized peaks at 22.6°, 23.8°, 28.3°, 33.0° and 33.9° indexed as the (002)_,_ (110), (102), (112) and (200) planes of α-WO_3_. The results of the cooling process, step by step from 900°C to room temperature at 50°C intervals as illustrated in figure 1*b*, clearly show that the WO_3_ sample was completely transferred from α-WO_3_ to β-WO_3_ phase at 700°C, and from β-WO_3_ to γ-WO_3_ at 150°C. A mild shift of transition temperature has been recognized which could be due to the over cooling effect.

**Figure 3.**
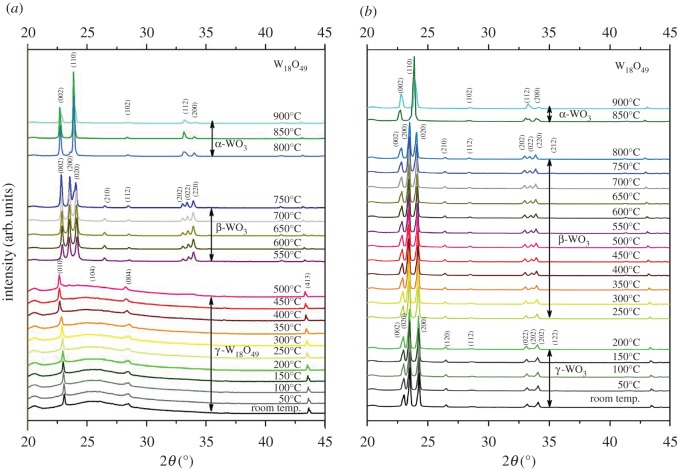
A series of *in situ* XRD profiles of the W_18_O_49_ nanowires obtained during heating (*a*) and cooling (*b*), from room temperature to 900°C, in 50°C increments.

The as-prepared W_18_O_49_ nanowires exhibited typical diffraction peaks at 23.5°, 26.2°, 28.1° and 43.6° at room temperature, which were indexed to the (010), (104), (004) and (413) planes of the monoclinic γ-W_18_O_49_ phase (COD 1528166) (figure 3*a*). This phase appeared to be stable up to 500°C and then some small shoulder peaks emerged in the diffractogram, which matched well with the orthorhombic β-WO_3_ phase at 550°C (COD 2107312). The main diffraction peaks at 22.9°, 23.5°, 24.2°, 26.5°, 28.6°, 33.1°, 33.6° and 34.0° were identified as the (002), (200), (020), (210), (112), (202), (022) and (220) planes of the orthorhombic phase, respectively. The orthorhombic phase remained stable until 700°C and then started to change to the α-WO_3_ tetragonal phase at 750°C. As shown in figure 3*a*, the 33.1° peak (202) started to merge with the 33.6° peak (022), and the 23.5° peak (200) started to merge with the 24.2° peak (020), when the temperature reached 750°C. At 800°C, peaks at 22.6°, 23.8°, 28.3°, 33.0° and 33.9° were identified as the (002), (110), (102), (112) and (200) planes of the α-WO_3_ tetragonal phase (COD 1521532), indicating the complete phase transition.

For the reverse cooling process, we have noted two key phase transitions based on the series of XRD profiles: from α-WO_3_ to β-WO_3_ at 800°C and β-WO_3_ to γ-WO_3_ at 200°C, as shown in figure 3*b*. However, the cooling process was more complicated than the heating stage, as we know that during heating the W_18_O_49_ nanowires are stable only up to 450°C, and that they will be fully oxidized, due to the minute residue oxygen in the low vacuum, to form β-WO_3_ at 500°C and transition to different phases until 900°C, as described earlier. Therefore, the cooling phase of the materials cannot be reversed back to W_18_O_49_ in composition even at room temperature. Furthermore, there would be some irreversible morphological changes upon heating at higher temperatures, which will be discussed later.

Our *in situ* XRD result of the WO_3_ nanoparticles was analogous to that reported by both Boulova & Lucazeau [25] and Lu *et al*. [18] in the lower temperature range, which was that the γ-WO_3_ phase started to transition to the β-WO_3_ phase at about 250°C. However, the α-WO_3_ phase transformation at about 670°C reported by them occurred at about 750°C in our case, which is much closer to the transition temperature of bulk WO_3_ structure [11]. Furthermore, we could not identify the triclinic (δ-WO_3_) and hexagonal (h-WO_3_) phases during our investigation. For the W_18_O_49_ nanowires, the present results further confirmed the previous *ex situ* study conducted by Sun *et al*. [19] that found the monoclinic γ-W_18_O_49_ only remained stable at temperatures below 450°C and completely transformed to the monoclinic β-WO_3_ phase above 500°C. However, these converted WO_3_ nanoparticles remained stable up to 900°C without further crystalline transitions based on the XRD results, which was slightly different from the original WO_3_ nanoparticles (lower by about 50°C than the nanowires). The result might be due to the difference in geometry and crystalline structures between the W_18_O_49_-converted WO_3_ and the as-received WO_3_, because the transformation from W_18_O_49_ to WO_3_ would inevitably involve crystal lattice rearrangement via atomic diffusion which will subsequently exaggerate the morphology evolution and oxygen vacancy filling [26]. WO_3_ presented the reversible phase transitions at lower temperature during cooling, compared with the heating process, whereas the W_18_O_49_ did not show any phase transition at low temperature (below 500°C), only existed as monoclinic, and did not exhibit reversible phase changes during cooling down from high temperature at 900°C (remained as WO_3_).

**Figure 4.**
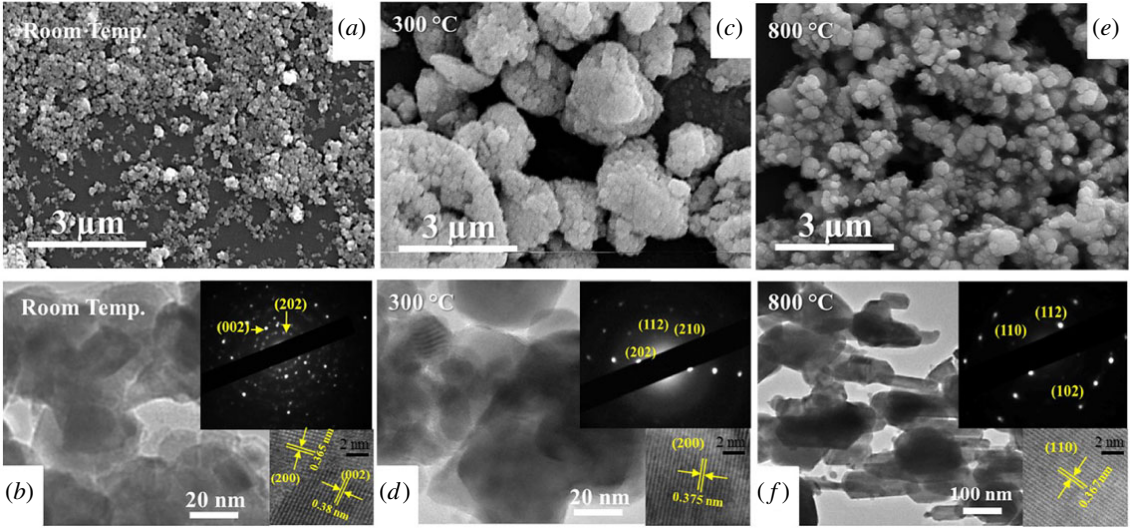
*Ex situ* SEM and HRTEM images of the WO_3_ nanoparticles after treatment at various temperatures: (*a*,*b*) room temperature, (*c*,*d*) 300°C and (*e*,*f*) 800°C. The insets show the diffraction patterns and lattice fringe images of the monoclinic (*γ*), orthorhombic (*β*) and tetragonal (*α*) phases of WO_3_.

**Figure 5.**
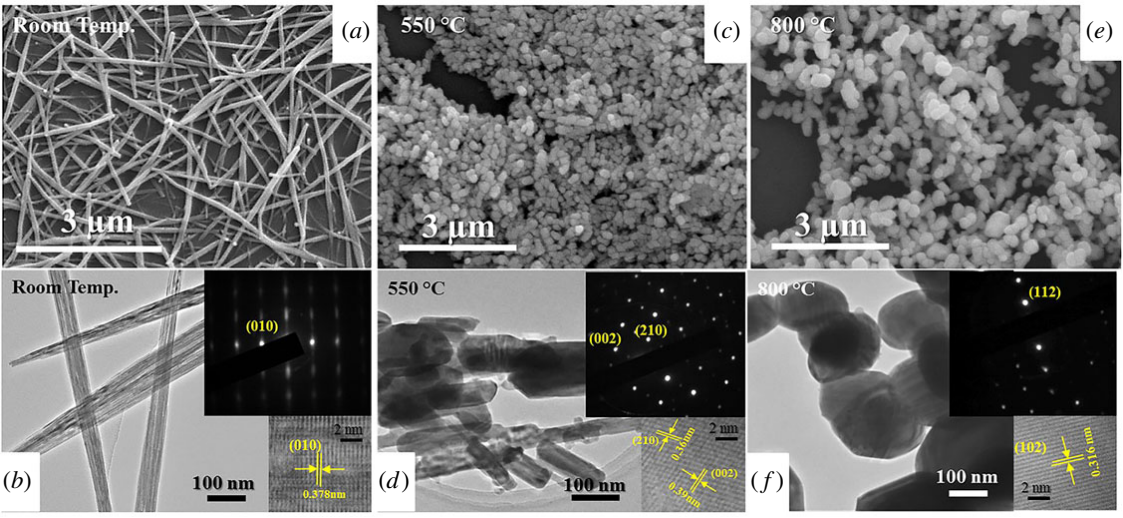
SEM and HRTEM images of the bundled W_18_O_49_ nanowires at room temperature (*a*,*b*), 550 (*c*,*d*) and 800°C (*e*,*f*), which were identified as the monoclinic γ-W_18_O_49_, orthorhombic β-WO_3_ and tetragonal α-WO_3_ phases, respectively.

The SEM and high-resolution TEM images both confirmed the morphological changes and phase transitions of the two materials, as shown in figures 4 and 5. The average size of the original monoclinic WO_3_ nanoparticle was about 40 nm in diameter (figure 4*a*), which appeared to be larger and severely agglomerated after the 300°C heating treatment (figure 4*c*). The nanoparticles were also no longer in the monoclinic γ-phase, having been converted to orthorhombic β-WO_3_. As the temperature was raised above 800°C further changes to both the crystal phase and morphology were observed, with tetragonal α-WO_3_ nanorods possessing diameters around 100–150 nm afforded. The HRTEM lattice fringes and the selected area diffraction patterns of each phase shown (figure 4, insets) matched very well with our XRD results.

The SEM images of the as-prepared W_18_O_49_ nanowires indicated average dimensions of approximately 3 µm in length and 50 nm in diameter. TEM analysis further confirmed that the as-prepared W_18_O_49_ nanowires consisted of ultrathin nanowires of only approximately 2–5 nm in diameter and up to 2 µm in length, self-assembled into bundles [19], hence the larger apparent diameter under SEM was owing to the lower resolution. In figure 5*b*, the streaking feature noted in the SAED pattern and the parallel HRTEM lattice fringes of the W_18_O_49_ nanowires both clearly demonstrated the bundled nature of the individual nanowires and that nanowires within a bundle were grown along the same direction of ⟨010⟩. The easily recognizable (010) plane spacing was approximately 0.378 nm, in good agreement with the XRD result of the monoclinic W_18_O_49_. After the 550°C treatment (figure 5*d*), short nanobricks, with a diameter of about 100 nm and length up to 200 nm, of WO_3_ were observed, which were subsequently converted into larger particles of about 200 nm in diameter after treatment at 800°C (figure 5*f*). The SAED and HRTEM results also showed that both the 550 and 800°C treated samples matched well with our XRD results. The ultrathin W_18_O_49_ sub-stoichiometric nanowires were only stable up to 500°C, then began to oxidize and were completely converted to the orthorhombic phase of WO_3_ at 550°C, due to the presence of minute residue oxygen in the low vacuum. Furthermore, the blue thin film turned to yellow during this stage. Meanwhile, the long and thin bundles were broken and reassembled into short and fat nanorods and eventually became much larger particles.

**Figure 6.**
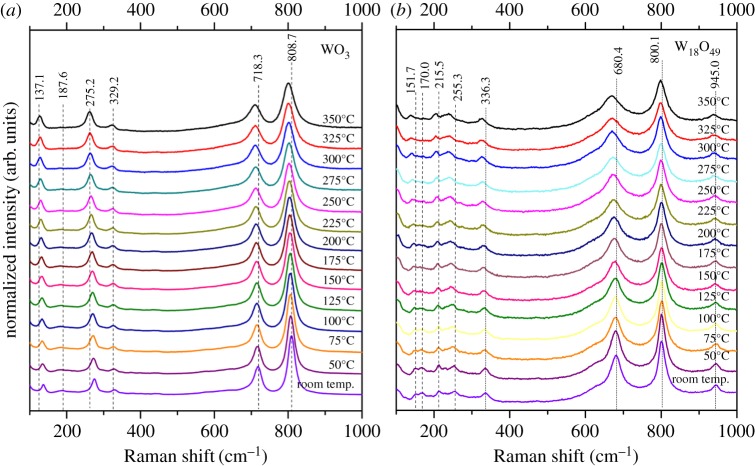
Raman spectra of the WO_3_ nanoparticles (*a*) and bundled W_18_O_49_ nanowires (*b*), under different annealing temperatures ranging from room temperature up to 350°C, increasing at 25°C for each stage, acquired with 1% laser power (0.336 mW).

While the above analytical techniques are extremely helpful in analysing the crystalline and morphological features, *in situ* Raman spectroscopy can provide more insight into the bonding within the two nanomaterials under examination. The *in situ* Raman spectra of the WO_3_ nanoparticles and the bundled W_18_O_49_ nanowires from room temperature up to 350°C (due to the limitation of the heating stage) were shown together in figure 5, for comparison. At room temperature, the structure of the monoclinic WO_3_ crystal consisted of corner-shared octahedral with the W atoms displaced from the centres, to form zigzag chains with alternating short and long W–O bond lengths. In figure 6*a*, the bands at 718 and 809 cm^−1^ were assigned to O–W–O stretches, associated with longer (1.88 Å) and shorter (1.82 Å) W–O bonds, respectively. The bands at 275 and 329 cm^−1^ were attributed to the O–W–O bending and O–W–O deformation modes, respectively. The other two peaks located at 137.1 and 187.5 cm^−1^ belonged to the lattice vibration modes, consistent with previous studies [27]. The structure of W_18_O_49_ could be derived from that of WO_3_ by introducing oxygen vacancies compensated by a pair of pentagonal columns of edge-sharing octahedral to produce hexagonal channels which run through the structure. This complex structure was expected to contain a range of O–W–O bond lengths which resulted in a broadening of the bands. The Raman spectra of the bundled W_18_O_49_ nanowires (figure 6*b*) exhibited three main regions. The high wavenumber bands included two peaks at approximately 680 and 800 cm^−1^, which could be assigned to the asymmetric and symmetric stretching vibration mode of O–W–O. The 255 cm^−1^ band was attributed to the O–W–O bending mode of bridging oxygen, and the 336 cm^−1^ band to the O–W–O deformation mode [28]. Moreover, the weak band at approximately 940 cm^−1^ could be used as the characteristic shift for W_18_O_49_ nanowires, because it did not exist in the WO_3_ nanoparticles, and has been ascribed to the W=O stretching vibration mode of a terminal oxygen. Such a moiety did not exist in WO_3_, but was expected to be present in the channels of sub-stoichiometric tungsten oxide species. The intensity of this peak remained constant during the heating experiment, indicating the stability of the nanowires up to 350°C. To clearly show the features of the Raman shift, we summarized the Raman peak positions in table 1. The decrease in the wavenumber of the stretching and bending modes with increasing temperature corresponded to an increase in the O–W–O bond length for both structures.

**Table 1:** The Raman shifts of WO_3_ and W_18_O_49_ in the range 100–1000 cm^−1^. *ν*, stretching; *δ*, deformation/in-plane bending.

		band positions (cm^−1^)						
		peak 1	peak 2	peak 3	peak 4	peak 5	peak 6	peak 7
	temperature (°C)	lattice modes		*δ* (O–W–O)		*ν* (O–W–O)		*ν* (W=O)
WO_3_ nanoparticles	room temp.	137.1	187.6	275.2	329.2	718.3	808.7	—
	50	134.7	184.8	272.5	327.6	716.6	807	—
	75	134.2	183.2	272.2	326.9	715.6	806.4	—
	100	133.5	184.9	270.0	325.6	714.9	805.8	—
	125	133.3	184.1	269.5	325.8	714.3	805.3	—
	150	132.6	183.9	269.3	325.5	714.1	804.9	—
	175	131.3	183.0	269.5	325.1	713.8	804.9	—
	200	131.3	184.2	266.9	325.1	713	803.8	—
	225	129.8	186.8	266.2	325.3	712.6	802.5	—
	250	129.1	181.8	265.6	324.6	711.8	802.5	—
	275	129.0	—	265.0	323.4	712.1	802.9	—
	300	127.9	—	263.6	323.5	710.6	801.8	—
	325	128.7	—	262.6	323.4	711	800.2	—
	350	127.1	—	262.1	322.6	709.8	800	—
	reverse at room temp.	129.3	181.4	267.3	322.3	711.8	802.3	—
bundle W_18_O_49_ nanowires	room temp.	151.7	170.0	255.3	336.3	680.4	800.1	945.0
	50	150.1	166.4	253.5	334.5	678.7	800.1	945.0
	75	153.6	166.4	248.1	334.5	680.4	801.8	948.3
	100	148.1	170.0	248.1	334.5	680.4	801.8	945.0
	125	148.1	170.0	246.3	332.7	678.7	800.1	943.3
	150	151.7	—	244.5	329.1	678.7	801.8	941.6
	175	146.3	—	244.5	330.9	675.3	801.8	945.0
	200	146.5	—	244.5	332.7	675.3	801.8	941.6
	225	146.3	—	240.9	330.9	675.3	798.4	943.3
	250	140.8	—	244.5	329.1	670.1	798.4	941.6
	275	144.4	—	239.1	325.5	671.8	800.1	938.3
	300	142.6	—	235.4	325.5	670.1	798.4	940.0
	325	138.9	—	240.7	325.5	671.8	798.4	936.6
	350	138.9	—	239.1	327.3	670.1	798.4	938.3
	reverse at room temp.	144.4	—	246.3	327.3	673.6	795.1	938.3

Boulova & Lucazeau [25] also studied the structural transitions of WO_3_ nanoparticles (average size approx. 35 nm) by using *in situ* Raman spectroscopy, from room temperature to 677°C (950 K). They found that samples began to transform from γ-WO_3_ to β-WO_3_ phase at a temperature of about 500 K (227°C) and then to α-WO_3_ at about 850 K (577°C). Similar phase transitions of much larger WO_3_ nanowires (40–80 nm in diameter and 1 µm in length) were reported by Lu *et al*. [18] using *in situ* Raman spectroscopy, where it was reported that the γ- to β-WO_3_ transition occurred at 230°C. Although these two studies did not provide the exact characteristic wavenumbers of β-WO_3_, our present wavenumber downshifts with increased heating temperatures appeared to agree well with their analyses. Downshifting to lower wavenumbers in the stretching and bending shift positions was a result of increased bond lengths between the W and O in the lattice. In fact, this increase in bond length was maintained, even back to room temperature, as verified in our *ex situ* HRTEM examination that the lattice distance of the (200) plane changed from 0.365 to 0.375 nm (figure 4*b*,*d*). Taking into account our *in situ* XRD results, we understood that the transition from the γ-WO_3_ to the β-WO_3_ phase occurred at around 300°C for the WO_3_ particles and at 550°C for the W_18_O_49_ nanowires. This *γ* to *β* transition point matched with our *in situ* Raman spectroscopy result that occurred at 275°C. Therefore, the disappearance of the 187.6 cm^−1^ band of WO_3_ was believed to be the fingerprint of the transition from γ- to β-WO_3_. Hence in table 1, we assigned the major stretching vibrational modes of β-WO_3_ phase at 802.9 and 712.1 cm^−1^, 323.4 and 265 cm^−1^ for the bending modes and 129 cm^−1^ for the lattice mode. For the W_18_O_49_ nanowires, we believed that they remained as the monoclinic γ-phase at 350°C, based on the two *in situ* observations; however, downshift with increase of temperature in the Raman spectra was noted. Thus, we believed that the peak shift could be an indicator of the relaxation of the channel inside the WO_3_ and W_18_O_49_ structures or the elimination of the impurity inside the structures [29]. Finally, it is clear that the phase change loop of these two different structures was different, due to the combination of the original geometry and slight compositional differences of the samples.

## 4. Conclusion

We have demonstrated that different geometries of WO_3_ nanoparticles and W_18_O_49_ nanowires exhibited different phase transition behaviours. The hot-stage XRD results have confirmed that, different from the γ-WO_3_ nanoparticles that converted to β-WO_3_ just below 300°C, the γ-W_18_O_49_ nanowires remained stable up to 500°C, then completely oxidized and transferred to β-WO_3_ at 550°C. The *in situ* Raman spectroscopy investigations have confirmed the downshift of peak position, which has been attributed to the increased length of the W–O chemical bonds inside the lattice. We have identified the 187.6 cm^−1^ fingerprint band as a means of identifying the phase transition from γ- to β-WO_3_ nanoparticle structures at 275°C, which is about 75°C lower than the relaxation temperature of above 350°C for the monoclinic γ-W_18_O_49_ nanowires. This finding suggests the better thermal stability and often higher performance of the ultrathin W_18_O_49_ nanowires compared with those of the WO_3_ nanoparticles. The understanding of these fine differences in phase transition and structural stability between bundled ultrathin W_18_O_49_ nanowires and spherical WO_3_ nanoparticles offers helpful guidance in the design and development of WO_x_-based nanomaterials in nanodevices.

## Data accessibility

This article does not contain any additional data.
